# Supplementary material for: Pushing the boundary of quantum advantage in hard combinatorial optimization with probabilistic computers
Source: Nat Commun. 2025 Oct 16;16:9193. doi: 10.1038/s41467-025-64235-y (PMC12533262; doi:10.1038/s41467-025-64235-y)
Supplement: Supplementary file 1 — Supplementary Information [file 41467_2025_64235_MOESM1_ESM.pdf]

Supplementary Information

Pushing the Boundary of Quantum Advantage in Hard Combinatorial Optimization with Probabilistic Computers

Shuvro Chowdhury, Navid Anjum Aadit, Andrea Grimaldi, Eleonora Raimondo, Atharva Raut, P. Aaron Lott, Johan H. Mentink, Marek M. Rams, Federico Ricci-Tersenghi, Massimo Chiappini, Luke S. Theogarajan, Tathagata Srimani, Giovanni Finocchio, Masoud Mohseni and Kerem Y. Camsari

I. DISCRETE-TIME SIMULATED QUANTUM ANNEALING (DT-SQA) ALGORITHM

The DT-SQA algorithm used in Fig. 2 of the main text relies on the Suzuki-Trotter approximation, where the partition function, Z_Q of a quantum Hamiltonian,

$$H_\mathrm{Q} = -\sum_{i<j} J_{ij}\sigma_i^z\sigma_j^z - \Gamma_x\sum_i \sigma_i^x \tag{S.1}$$

with σ_i^α ($\alpha \in \{x, y, z\}$) being Pauli spin matrix at site i, is approximated by the partition function, Z_C of a classical Hamiltonian, H_C as

$$Z_\mathrm{Q} = \mathrm{tr}\left[\exp\left(-\beta H_\mathrm{Q}\right)\right] = \lim_{R\to\infty} Z_\mathrm{C} = \lim_{R\to\infty}\exp\left(-\beta H_\mathrm{C}\right) \tag{S.2}$$

where H_C, corresponding to H_Q, is defined as

$$H_\mathrm{C} = -\sum_{k=1}^{R}\left[\sum_{i<j} J_{\parallel,ij}\sigma_{i,k}\sigma_{j,k} + \sum_i J_\perp\sigma_{i,k}\sigma_{i,k+1}\right] \text{ with } J_{\parallel,ij} = \frac{J_{ij}}{R} \text{ and } J_\perp = -\frac{1}{2\beta}\ln\left[\tanh\left(\frac{\beta\Gamma_x}{R}\right)\right]. \tag{S.3}$$

where $\sigma_{i,j}$ (without any superscript) denotes Ising spin ($\sigma_{i,j} \in \{-1, +1\}$) at site i of replica j. Thus, a d-dimensional quantum Hamiltonian can be theoretically mapped to a $(d+1)$-dimensional classical Hamiltonian, where the extra dimension corresponds to the replica dimension. When R approaches infinity, the partition function corresponding to the quantum Hamiltonian is exactly reproduced by the partition function of the mapped classical counterpart. However, for practical purposes or when DT-SQA is used as a classical algorithm (for example, in optimization), a finite number of replicas typically in the range of 10 to 100–may be preferred [1–3]. A pseudocode outlining our implementation of the DT-SQA algorithm is presented in Algorithm S1. As described in the Methods section, graph coloring is used to partition the spin system into independent sets that can be updated in parallel. Each color corresponds to one of these independent sets. Algorithm S1 implements this update scheme by looping over colors.

There is also a continuous-time version of the DT-SQA algorithm (CT-SQA) [4]. However, CT-SQA lacks the straightforward hardware implementation offered by DT-SQA. Although both algorithms emulate the equilibrium statistics of the quantum system, differences arise when comparing their transient dynamics to those of a quantum annealer.

A. Residual energy as a function of total Monte Carlo effort

In the main text, we analyze the scaling behavior of the residual energy as a function of Monte Carlo sweeps for three distinct Discrete-Time Simulated Quantum Annealing (DT-SQA) systems, each characterized by a different number of Trotter slices. We define a Monte Carlo sweep as the process of updating every spin in the system exactly once. Consequently, larger systems naturally involve more computational effort, as they necessitate a greater number of probabilistic spin flips per sweep.

This definition aligns intuitively with our hardware-centric context. We envision the different systems, each comprising R replicas, as separate black-box computational units or stand-alone chips. As previously detailed in Refs. [5–8], our graph-colored architecture is designed to mimic a truly asynchronous analog probabilistic annealer (one example being a stochastic Magnetic Tunnel Junction based probabilistic computer [9]) and as such it enables updates across the entire network in constant time.

This uniform update time primarily depends on the topology of the network rather than system size. Specifically, if a given graph has degree scaling as $\mathcal{O}(k)$, where k denotes the number of neighbors per node (as in the case of 3D spin glasses considered in this work), the clock frequency that sets the update frequency is mainly determined by the time required to compute the local field as given by Eq. (5), a calculation independent of the overall network size, as we demonstrate in Supplementary Fig. S11 and Supplementary Table S1.

Our use of Monte Carlo sweeps for systems of different sizes is different from how Monte Carlo effort is defined in CPU-centric comparisons [10]. As carefully discussed in Ref. [10], an analog quantum annealer acquires an $\mathcal{O}(N)$ speedup (similar to our asynchronous architecture) due to its linearly growing resources with problem size and this parallel speedup must be separated from intrinsically quantum speedups. Given that our systems also benefit from the same speedup, our definitional choice for MCS is appropriate.

Nevertheless, it is crucial to note that our scaling argument against quantum annealing does not hinge upon any MCS definition, since the residual energy comparisons are made on a fixed size, hence N does not vary. As demonstrated in Fig. S1, the residual energy exhibits a clear power-law dependence on annealing time or Monte Carlo sweeps. Thus, multiplying the annealing duration by a constant factor (in this case, the number of replicas R) does not alter the fundamental slope of the scaling:

$$\rho_{\mathrm{E}}^{\mathrm{f}}(Rt_{\mathrm{a}}) \propto (Rt_{\mathrm{a}})^{-\kappa_{\mathrm{f}}} = (\mathrm{constant})\, t_{\mathrm{a}}^{-\kappa_{\mathrm{f}}}. \tag{S.4}$$

B. Performance of DT-SQA algorithm on embedded instances

In Fig. 2 of the main text, we presented scaling results for logical instances using the DT-SQA algorithm. Fig. S2 shows the scaling results from DT-SQA experiments on embedded instances. As in logical instances, the slope increases gradually with the number of replicas for the embedded instances, however, achieving the same slope as the quantum annealer requires more replicas compared to logical instances. Also, the embedded instances as provided require four colors for graph coloring (due to the embedding requirements). An even number of replicas are used so that the replicated networks of the embedded instances can also be colored with four colors.

C. Cube size and Trotter replica dependence of DT-SQA

Fig. S3 illustrates the dependence of the final residual energy on annealing time (t_a) for different cube sizes (L) of logical instances. The slopes (absolute value) of the plots decrease as L increases, but all plots eventually reach a flat plateau region. This behavior is consistently observed across different numbers of Trotter replicas (R) as shown in the figure. The residual energy at which the plateau occurs decreases with an increasing number of Trotter replicas, also observed in [3].

II. ANALYSIS OF DT-SQA SCALING USING EXTREME VALUE THEORY

In this section, we explain the increase in DT-SQA slopes with increasing Trotter replicas using extreme value theory (EVT). Recall that in our DT-SQA simulations, we have R interconnected Trotter replicas and we select the replica with the best

Algorithm S1: Discrete-time simulated quantum annealing algorithm with p-computers

Input: Weights, biases, number of replicas, number of sweeps, colormap, annealing schedule, temperature
Output: State corresponding to minimum energy, m_{opt}

```
Function p-computer(weights, biases, colormap, temp.):
    for each color in the colormap do
        for each p-bit in the color do
            solve Eq. (5) and Eq. (6).
Divide weights by number of replicas.
Generate all replicas as indicated in number of replicas.
Insert transverse couplings between the replicas.
Perform graph coloring for the replicated network given the colormap of a single replica.
Initialize all spins in all replicas to random states.
for each sweep until number of sweeps is reached do
    Get the transverse field value from the annealing schedule.
    Update the transverse coupling using the transverse field.
    Sample p-bit states from p-computer.
Compute energy of each replica.
return p-bit states for the replica with the minimum energy.
```

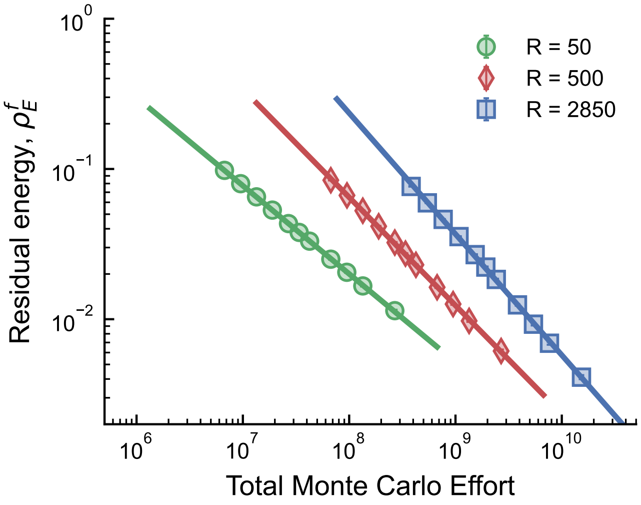

FIG. S1. **Slope of the residual energy, ρ_E^f as a function of total Monte Carlo effort:** The residual energy is re-plotted against the total Monte Carlo effort for the logical instances of size $15 \times 15 \times 12$. Here total Monte Carlo effort is defined as the total number of attempted spin flips during the whole annealing process. We emphasize that doing so only changes the prefactor and does not change the slope of the plots because of the power-law nature since multiplying time with any constant factor does not change the slope. We also emphasize that the Total Monte Carlo effort is a fixed-size CPU-centric measure, as discussed in Ref. [10], not appropriate in our context, as we discuss in the text.

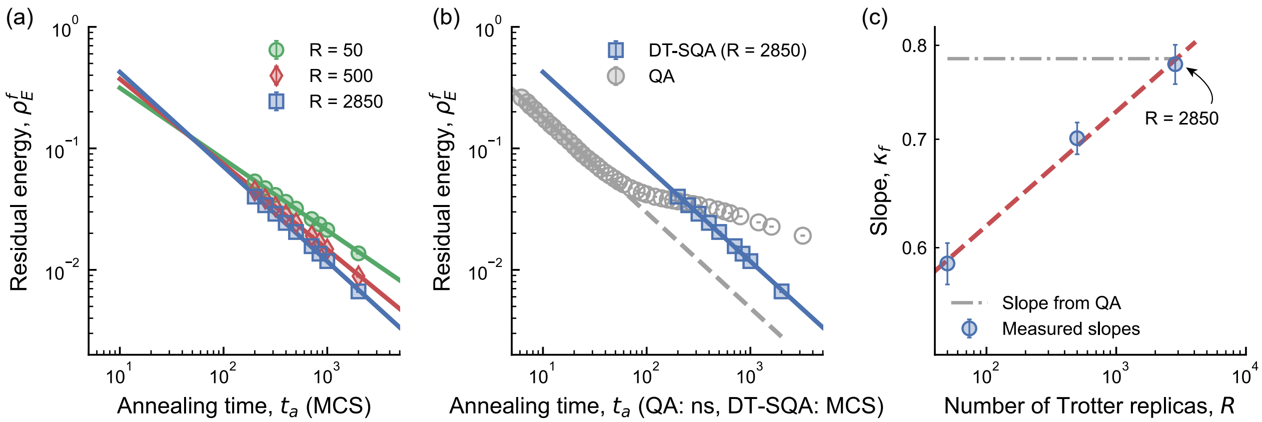

FIG. S2. **Replica scaling of the DT-SQA on embedded instances:** (a) Residual energy, $\rho_{\mathrm{E}}^{\mathrm{f}}$, plotted as a function of the annealing time, t_a, for embedded instances of size $15 \times 15 \times 12 \times 2$ (the latter 2 represents the number of physical qubits used to represent a logical spin), for three distinct values of R. In these instances, each lattice point consists of a pair of spins strongly coupled by a ferromagnetic interaction with an absolute strength of 2. (b) The DT-SQA curve with $R = 2850$ in (a) is compared to the slope obtained from a quantum annealer, showing nearly identical slope as reported in Ref. [11]. (c) The measured slopes (κ_{f}) from DT-SQA simulations are plotted against the number of replicas, R. The red dashed line serves as a visual guide, while the gray dotted-dashed line represents the slope derived from the QA. The slope for the quantum annealer can be matched by using more than 2850 replicas. Error bars denote 95% confidence interval of fitting.

(minimum) energy. The interconnection of Trotter replicas complicates the direct application of EVT. Therefore, we first describe the conventional EVT, followed by the modified EVT for interconnected Trotter replicas.

Conventional EVT: A straightforward application of EVT can be demonstrated with the following experiment: we run P independent copies of DT-SQA simulations, each consisting of R Trotter replicas. From each of these P independent copies, we select the minimum energy replica and then choose the minimum among these P minimum energies. Since these P minimum energies are independent and identically distributed, their distribution is approximately a Gaussian distribution, particularly at low MCS as shown in Supplementary Fig. S4. At high MCS, the distributions become skewed to the left, due to the hard constraint imposed by the ground state which serves as a lower bound.

For the derivations that follow, we assume a Gaussian distribution for the energies. Let $E_1, E_2, \ldots, E_P$ represent the energies of P independent and identically distributed (i.i.d.) Gaussian random variables, each characterized by a mean μ and standard deviation σ. Our objective is to derive the expected value of the minimum of these P runs, $\mathbb{E}\left(\min\left(E_1, E_2, \ldots, E_P\right)\right)$ which

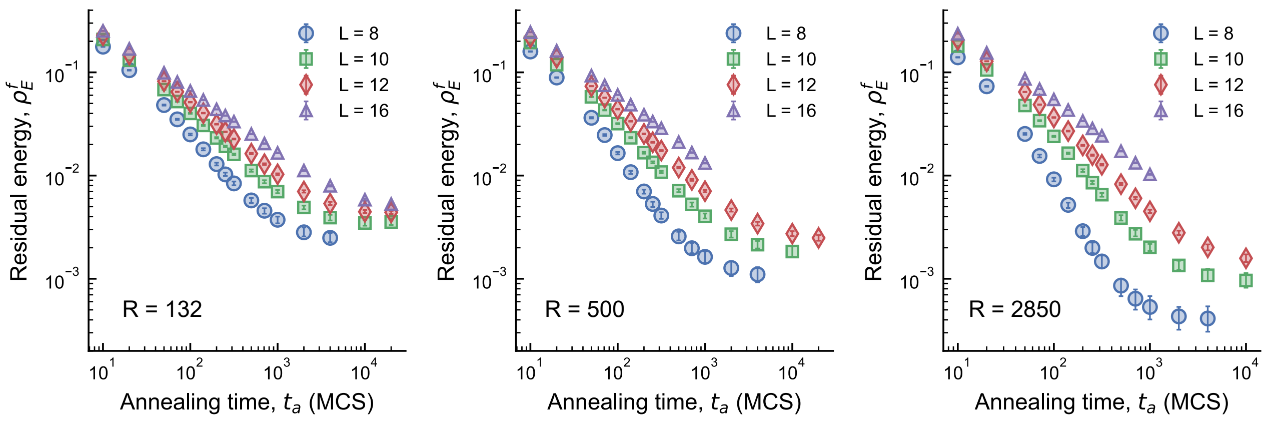

FIG. S3. **Cube size and replica dependence of DT-SQA:** Residual energy ($\rho_{\mathrm{E}}^{\mathrm{f}}$) is plotted as a function of annealing time (t_{a}) for various cube sizes (L) and three different number of Trotter replicas (R). Each data point represents an average over 300 problem instances, with each instance averaged over 50 independent runs. Error bars represent the 95% bootstrap confidence interval of the mean across 300 spin-glass instances.

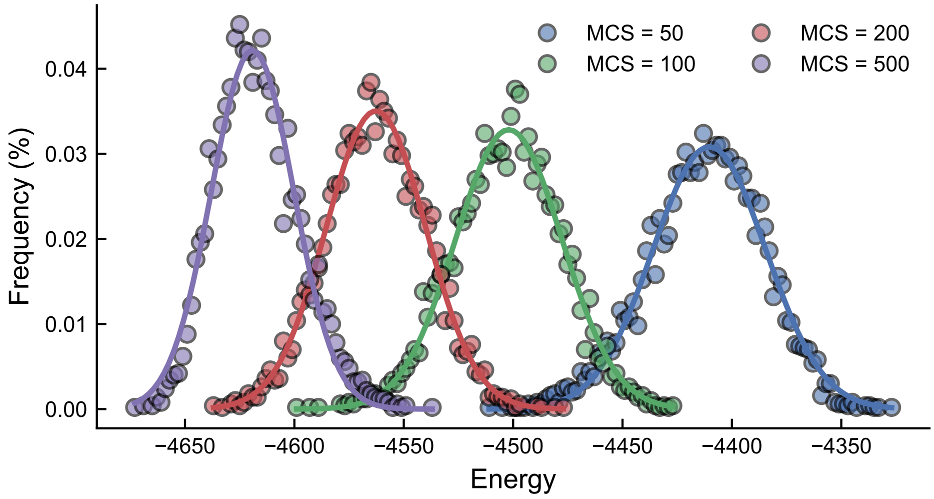

FIG. S4. **Gaussian distribution of minimum energy values:** The distribution of minimum energies for a randomly chosen spin-glass instance is shown at various MCS values, based on 5000 independent runs. In each run, DT-SQA is performed with 32 Trotter replicas for annealing at a fixed MCS, and the best energy replica is selected at the end of the annealing process. The distributions closely follow a Gaussian shape for smaller MCS values, as indicated by the dashed lines representing Gaussian fits. At higher MCS values, the distributions become increasingly skewed to the left due to the hard constraint imposed by the ground state energy.

plays a crucial role in improving the residual energy and, consequently the slope in DT-SQA simulations. The probability density function (PDF) of a Gaussian random variable E_i is given by:

$$f\left(x\right)=\frac{1}{\sqrt{2\pi}\sigma}\exp\left(-\frac{\left(x-\mu\right)^2}{2\sigma^2}\right) \tag{S.5}$$

The cumulative distribution function (CDF) of a random variable E represents the probability that E takes a value less than or equal to a specific value x. Mathematically, the CDF, $F(x)$, is defined as:

$$F(x)=\Pr\left(E\leq x\right) \tag{S.6}$$

For a continuous random variable, the CDF can be obtained by integrating its probability density function (PDF) $f(x)$:

$$F(x)=\int_{-\infty}^{x} f(x)\,\mathrm{d}x \tag{S.7}$$

The CDF satisfies the property that

$$\lim_{x\to\infty} F(x) = 1 \tag{S.8}$$

which ensures that the total probability adds to 1. For a Gaussian random variable, the cumulative distribution function (CDF) is given by:

$$F(x) = \frac{1}{\sqrt{2\pi}\sigma}\int_{-\infty}^{x} \exp\left(-\frac{(x-\mu)^2}{2\sigma^2}\right) \mathrm{d}x = \frac{1}{2}\left[1+\mathrm{erf}\left(\frac{x-\mu}{\sqrt{2}\sigma}\right)\right] \tag{S.9}$$

where $\mathrm{erf}(\cdot)$ is the error function.

The CDF allows us to compute probabilities over intervals and is particularly useful to find the probability that the minimum of P variables falls below a given threshold. To calculate the probability that the minimum of these P random variables E_i is less than or equal to a certain threshold x, i.e., Pr(min $(E_1, E_2, \ldots, E_P) \leq x$), we start by noting that for a single random variable E_i, Pr(min $(E_i) \leq x$) = Pr($E_i \leq x$) and is given by its CDF. For two variables, E_1 and E_2, the probability that their minimum is less than or equal to x can be computed using their complement probabilities, similar to the Bernoulli case. Specifically,

$$\Pr(\min(E_1, E_2) \leq x) = 1 - \Pr(\min(E_1, E_2) > x) \tag{S.10}$$

But min $(E_1, E_2) > x$ implies that both $E_1, E_2 > x$ and therefore,

$$\Pr(\min(E_1, E_2) \leq x) = 1 - [\Pr(E_1 > x)\Pr(E_2 > x)] \tag{S.11}$$

Using the property Pr $(E_i > x) = 1 - \Pr(E_i \leq x) = 1 - F(x)$, this can be rewritten as:

$$\Pr(\min(E_1, E_2) \leq x) = 1 - \{[1-\Pr(E_1 \leq x)][1-\Pr(E_2 \leq x)]\} = 1 - [1-F(x)]^2 \tag{S.12}$$

Generalizing this result to P independent random variables, we obtain:

$$\Pr(\min(E_1, E_2, \ldots, E_P) \leq x) = F_P(x) = 1 - [1-F(x)]^P = 1 - 2^{-P}\left[\mathrm{erfc}\left(\frac{x-\mu}{\sqrt{2}\sigma}\right)\right]^P \tag{S.13}$$

where $\mathrm{erfc}(\cdot) = 1 - \mathrm{erf}(\cdot)$ is the complementary error function.

Finally, the expected value of the minimum of these P random variables can be found by calculating the mean of the PDF associated with this CDF. Specifically, the expected value is derived as:

$$\mathbb{E}(\min(E_1, E_2, \ldots, E_P)) = \int_{-\infty}^{+\infty} x\,\frac{dF_P(x)}{dx}\mathrm{d}x = \frac{2^{0.5-P}P}{\sqrt{\pi}\sigma}\int_{-\infty}^{+\infty} x\,\exp\left[-(x-\mu)^2/2\sigma^2\right]\left[\mathrm{erfc}\left(\frac{x-\mu}{\sqrt{2}\sigma}\right)\right]^{P-1}\mathrm{d}x \tag{S.14}$$

While Eq. (S.14) formally expresses the expected value of the minimum energy, it lacks a simple closed-form solution for $P > 5$ due to the complexity of integrating terms involving the complementary error function. However, we can obtain an excellent approximation by focusing on the median instead of the mean, leveraging the fact that for symmetric distributions like the Gaussian, the mean and median are close in value.

So, we proceed to solve for the median x_p such that $F_P(x_p) = 0.5$:

$$F_P(x_p) = 0.5 = 1 - 2^{-P}\left[\mathrm{erfc}\left(\frac{x_p-\mu}{\sqrt{2}\sigma}\right)\right]^P \tag{S.15}$$

which leads to the following solution:

$$x_p = \mu + \sqrt{2}\,\sigma\,\mathrm{erfc}^{-1}\big(2^{\frac{P-1}{P}}\big). \tag{S.16}$$

which is an exact expression that finds the median. However, this expression also does not reveal the relationship between x_p and P explicitly. In order to get a more revealing expression, we find an asymptotic expansion of x_p which leads to:

$$x_p \approx \mu - \sigma\sqrt{\ln\frac{2}{\pi(\ln 4)^2} + \frac{\ln 2}{P} + 2\ln P - \ln\left[\ln\frac{2}{\pi(\ln 4)^2} + \frac{\ln 2}{P} + 2\ln P\right]} \tag{S.17}$$

when $P \to \infty$,

$$x_p \approx \mu - \sigma\sqrt{2\ln P} \tag{S.18}$$

which is a textbook result from EVT. Thus, as P increases, the minimum energy decreases as $\sqrt{\ln P}$, which reflects how increasing the number of replicas enhances the system's ability to find lower-energy states.

The functional form of the final residual energy, $\rho_{\rm E}^{\rm f}$ can be obtained from the Supplementary Eq. (S.16) and the definition of $\rho_{\rm E}^{\rm f}$ from the main text:

$$\rho_{\rm E}^{\rm f}(t) = a(t) + \sqrt{2}\, b(t)\, \mathrm{erfc}^{-1}\left(2^{\frac{P-1}{P}}\right). \tag{S.19}$$

where $a(t)$ and $b(t)$ are two time-dependent parameters which are related to the average mean and average standard deviation of distributions of residual energies from individual runs at a fixed MCS. To validate our theory, we conduct P independent DT-SQA simulations, each with 32 Trotter replicas, after which we select the best solution across all P runs. The results of this experiment are shown in Fig. S5(a-c). We observe that with approximately $P \approx 50$ repetitions, the slope achieved matches the slope reported in [12].

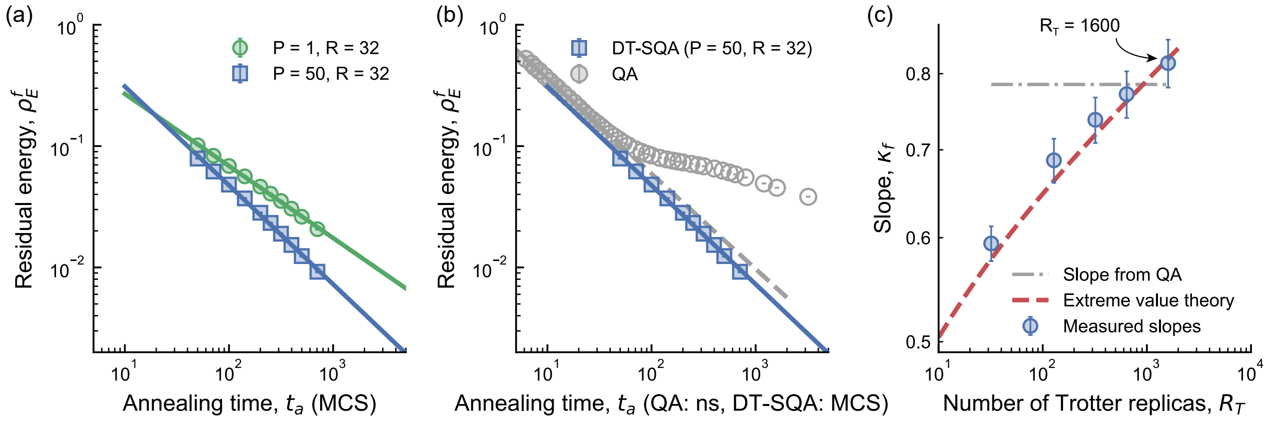

FIG. S5. **Enhanced scaling using independent runs of Trotter replicas in DT-SQA:** (a) Residual energy ($\rho_{\rm E}^{\rm f}$) as a function of annealing time (t_a) is shown for P independent DT-SQA simulations, each with 32 Trotter replicas. The best solution from these P experiments is selected. R_T is defined as the total number of Trotter replicas, and is equal to $R_T = 32P$. Results are shown for ($P = 1, R = 32$) and ($P = 50, R = 32$). Error bars denote the 95% bootstrap confidence interval of the mean across spin-glass instances. (b) Comparison of DT-SQA results with $R_T = 1600$ to those of the quantum annealer (QA). With $P \approx 50$, DT-SQA achieves a slope comparable to the slope reported for QA in [12]. (c) Slope ($\kappa_{\rm f}$) as a function of R_T. The red dashed line represents predictions from extreme value theory (EVT), while the gray dotted-dashed line corresponds to the slope from QA. Unlike the original DT-SQA algorithm, where all Trotter replicas are interconnected, the EVT-based approach achieves better scaling with fewer Trotter replicas. Error bars denote 95% confidence interval of fitting. The total number of replicas R_T should be compared with the total nuber of replicas R in Fig. 2 in main text. Fig. 2 uses only $P = 1$ which implies $R_T = R$, for simplicity we did not use R_T there. Error bars denote 95% confidence interval of fitting.

For each MCS value, the mean (μ) and standard deviation (σ) for each instance are obtained by fitting the distribution of the sampled best energies from multiple DT-SQA simulations (each simulation contains 32 Trotter replicas, and only the best energy replica is selected). The averages of these means and standard deviations are then computed across all 300 instances annealed at the same MCS. These averages are converted into residual energies using Eq. (2) in the main text. Residual energy predictions are derived from these mean and standard deviation values using Supplementary Eq. (S.19), as shown in Fig. S5. We find excellent agreement between experimental results and predictions from conventional EVT as shown in Supplementary Fig. S5(c).

Modified EVT: The extreme value theory (EVT) predictions for Fig. 2(c) are more involved and not exact. Two technical challenges arise: (1) all Trotter replicas are interconnected, so the block size is not known a priori, unlike the conventional EVT approach and (2) the block size may depend on the MCS values through Γ_x and $J_\perp$. The latter stems from the fact that the correlation length along the replica direction varies with MCS, as shown in Fig. S6. Despite these challenges, we apply EVT for an approximate understanding. We formulate a self-consistent approach as follows: we start by guessing a block size and partitioning the total number of Trotter replicas accordingly. For each partition, the best energy among the Trotter replicas within that partition is determined. As before, the mean and standard deviation of these best energy values are computed for each instance at a given MCS and averaged over all 300 instances and multiple runs per instance. Using these average mean and standard deviation values, we predict the residual energy with Supplementary Eq. (S.19). This predicted residual energy is then compared with the actual residual energy computed using Eq. (2) in the main text. The procedure is repeated until a block size is found where the prediction and actual residual energies closely match, as shown in Supplementary Fig. S7. For low MCS

values, the block sizes determined from this approach approximately align with the correlation lengths obtained independently from simulations (see Supplementary Fig. S6). This alignment provides a justification for our modified EVT approach. At higher MCS values, however, the distributions deviate from a Gaussian shape, becoming increasingly skewed to the left. Consequently, deviations from Supplementary Eq. (S.19) become more pronounced.

Comparison between conventional and modified EVT: Despite the seeming similarities, the independent (conventional) and interconnected (modified) EVT approaches are not equivalent. Supplementary Fig. S8 shows that the conventional approach deviates from the power-law behavior, encountering an early flat plateau around 1000 MCS. This behavior aligns well with the correlation trends shown in Supplementary Fig. S6, where at around 1000 MCS the replica-to-replica correlation length exceeds the system size ($R = 32$) for the conventional EVT.

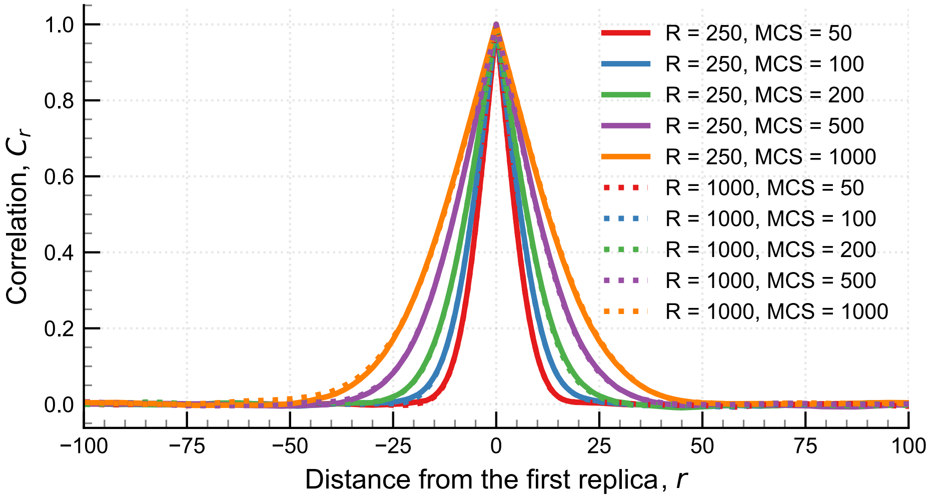

FIG. S6. **Decay of correlation length along the replica direction:** The average correlation ($C_r = (1/n)\sum_n \sigma_{i,0}\sigma_{i,r}$) between the replicas as a function of the distance (r) from the first replica ($r = 0$) is shown for the DT-SQA algorithm, with various total number of Trotter replicas R and annealing times (t_{a}, in MCS units). We measure C_r from the first replica, however, due to the periodic boundary conditions along the replica direction, C_r is measured to be invariant across all replicas. The correlation length along the replica direction shows a strong dependence on the annealing times (t_{a}) with longer annealing times leading to broader correlation peaks. However, the dependence on the number of Trotter replicas (R) is minimal, as indicated by the nearly overlapping dashed and solid lines for different R values.

III. FEASIBILITY ANALYSIS OF DT-SQA

As shown in Fig. 2 of the main text, a large number of replicas ($R = 2850$) is required for the DT-SQA algorithm to match the scaling exponent of the quantum annealer for 3D spin glass problems. We now evaluate the feasibility of having 2850 replicas on a single chip. This way, a hardware implementation of DT-SQA would physically house and update all replicas in parallel, unlike software-based simulations where replicas are updated sequentially on a CPU. The feasibility analysis we present here is equally applicable to the APT algorithm, which achieves performance on par with or exceeding DT-SQA and the quantum annealer while requiring fewer replicas (132 replicas as discussed in the main text) and further improvement in performance is expected with 2850 replicas. To assess the feasibility, we conduct a detailed physical design analysis. For our analysis, we use the open-source mflowgen physical design flow [13] with the open-source ASAP7 7 nm process design kit (PDK) [14]. Custom RTLs are developed to implement the algorithm based on the p-computing architecture shown in Supplementary Fig. S9, incorporating multi-phase clocking to manage timing across the design. Synthesis is carried out using Cadence Genus, followed by floorplanning, power distribution network (PDN) generation, clock tree synthesis (CTS), and final place-and-route (P&R) using Cadence Innovus. Fig. S10 provides examples of designs after placement and routing, with the corresponding design metrics summarized in Table S1. The routed designs are verified to meet timing constraints under a 3-phase clock (to allow provision for using odd number of replicas), with each phase operating at a frequency of 100 MHz. The final designs, completed after placement and routing, demonstrate that: (1) an ASIC implementation is feasible, and (2) the area required for this ASIC scales linearly with problem sizes.

Fig. S11 shows the area scaling of placed and routed designs for different instances of the DT-SQA algorithm. The area scaling follows a linear trend, with the largest design–featuring 13435 p-bits–occupying approximately 1 mm^2. Extrapolating this scaling to modern chip dimensions, a chip measuring 28.61 mm $\times$ 28.61 mm could accommodate approximately 7.66 million p-bits, corresponding to 2850 replicas of size $15 \times 15 \times 12$, achieving a scaling similar to that of a quantum annealer.

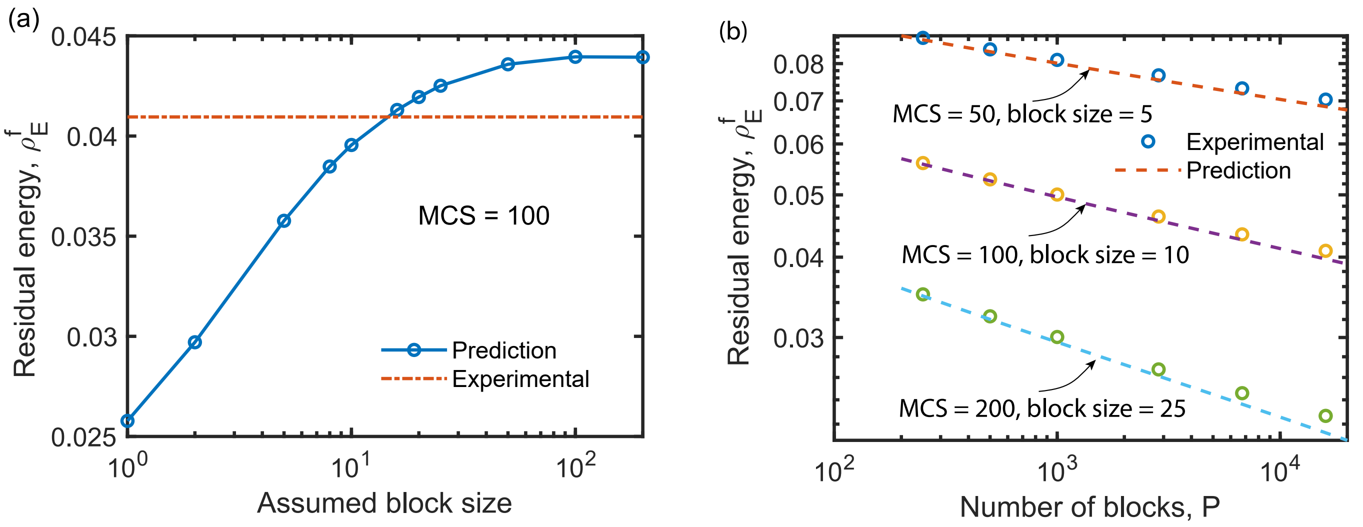

FIG. S7. **Comparison of predictions from the modified EVT with experimentally obtained residual energies:** (a) The block size extraction procedure based on extreme value theory (EVT) is illustrated for t_a = 100 MCS. Various block sizes are assumed, and the corresponding residual energies are predicted. The block size that yields a prediction closest to the experimental residual energy is selected. (b) The procedure in (a) is repeated for different MCS values (such as t_a = 50, 100 and 200 MCS). The chosen block sizes for each MCS value are shown alongside the number of blocks (P). The predictions from the modified EVT align closely with the experimental residual energies. These results are in approximate agreement with the correlation lengths shown in Supplementary Fig. S6.

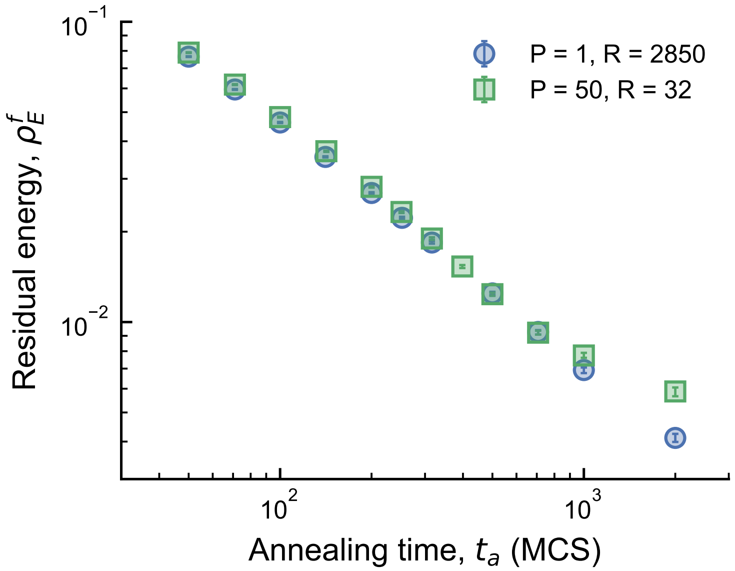

FIG. S8. **Comparison of two DT-SQA approaches used in this work:** For logical instances of size $15 \times 15 \times 12$, EVT based approach of DT-SQA ($R = 32$, $P = 50$) is compared against the conventional DT-SQA approach with $R = 2850$, $P = 1$. Both approaches share the same initial slope, which exceeds the quantum annealer's slope. However, the EVT-based approach reaches a flat plateau at a relatively higher residual energy emphasizing that the two approaches are not identical (see text). Error bars denote 95% bootstrapped confidence interval of mean over 300 instances.

IV. APT ALGORITHM WITH ISOENERGETIC CLUSTER MOVE (ICM)

Parallel tempering (PT) and its variants are a standard choice for solving challenging optimization problems such as the 3D spin glass. Hence, we also evaluate the performance of PT. Like DT-SQA, PT is also a replica-based algorithm. For our analysis, we employ an adaptive version of PT (APT) that includes a preprocessing step to determine the temperature schedule and optimize the number of replicas required. To further enhance the performance of APT, we incorporate isoenergetic cluster moves (ICM). A pseudocode detailing the adaptive parallel tempering algorithm, including the temperature schedule preprocessing and

TABLE S1. **Details of the physical flow designs:** Detailed information about the physical flow design performed in this study are listed. The design results are based on ASAP7 - a 7 nm finFET predictive process design kit (PDK) with mflowgen. L represents the dimension of the cube and R denotes the number of replicas used.

**Number of p-bits**	27	64	125	2687	5374	8061	10748	13435	7.66×10^6
**Cube dimension**, L	3	4	5	15	15	15	15	15	15
**Number of replicas**, R	1	1	1	1	2	3	4	5	2850
**Flow status**	routed	routed	routed	routed	routed	routed	routed	routed	projected
**Number of standard cells**	11303	27653	54685	1.385×10^6	2.978×10^6	4.886×10^6	6.562×10^6	8.199×10^6	4.7×10^9
**Timing met (per phase) MHz**	100	100	100	100	100	100	100	100	100
**Area (mm^2)**	0.001581	0.003844	0.007594	0.184317	0.382561	0.634809	0.851805	1.065527	818.34

ICM, is presented in Algorithm S2. Additional details regarding the exact parameters used can be found in the Methods section of the main text.

A. Temperature profile and swap acceptance rate in APT

We apply the preprocessing algorithm individually to each problem instance as detailed in the Methods section. The temperature profiles for all 300 instances of size $15 \times 15 \times 12$ are shown in Fig. S12. The profiles are highly consistent across instances, with only slight deviations in the low-temperature (high β) region. In principle, a temperature profile generated for a randomly selected instance could be applied to all instances without significantly impacting performance. However, in this work, we optimize the temperature profiles for each instance.

Additionally, Fig. S12 includes the standard deviation of the sampled Monte Carlo energies at each iteration (corresponding to each β). The standard deviation decreases monotonically as β increases, confirming that the replica temperatures are chosen appropriately.

Fig. S13 shows the typical swap acceptance rate in the APT algorithm for logical instances of size $15 \times 15 \times 12$. For optimal performance, it is generally recommended that the acceptance rate remains approximately constant. As shown, the acceptance rate stays roughly constant at around 40%, except for the last few replicas. However, since the algorithm selects the replica with the minimum energy at each swap, the lower acceptance rate of the last few replicas does not impact the overall performance of

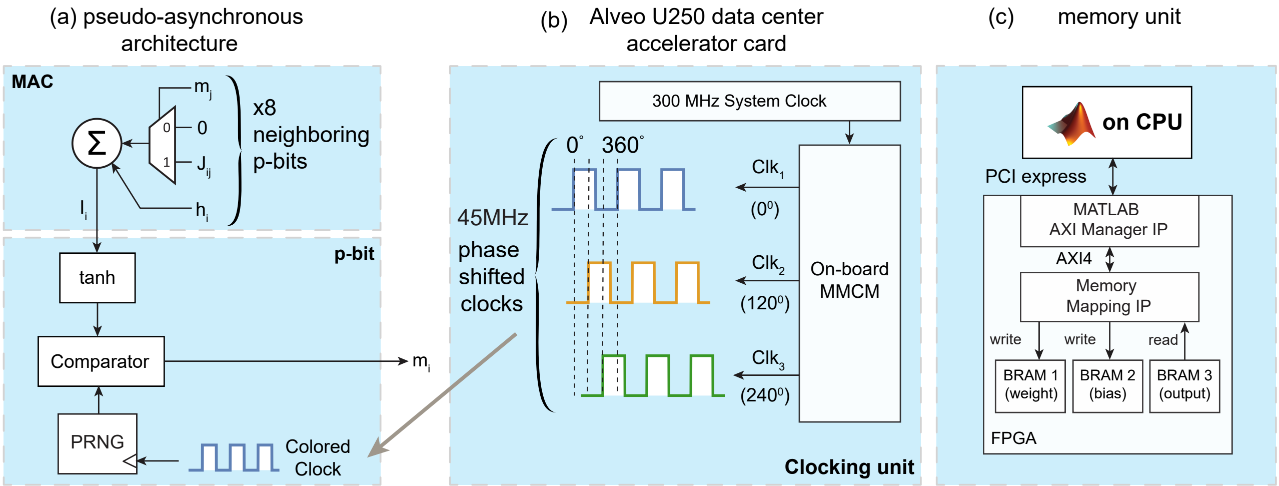

FIG. S9. **p-computing architecture used in the feasibility analysis of the DT-SQA algorithm:** (a) The pseudo-asynchronous architecture features a multiplier-accumulator (MAC) unit that implements Eq. (5). Each p-bit unit consists of a linear feedback shift register (LFSR)-based pseudorandom number generator (PRNG), a lookup table for the activation function (tanh), and a comparator to generate a binary output. (b) The clocking unit on the Alveo U250 data center accelerator card generates three phase-shifted clocks (0°, 120°, and 240°) at 45 MHz from a 300 MHz system clock using an on-board mixed-mode clock manager (MMCM). These are used to trigger the PRNGs inside the colored p-bit blocks. (c) The memory unit interfaces with the CPU via Peripheral Component Interconnect (PCI) Express. Data transfer between MATLAB and the FPGA is managed through Advanced eXtensible Interface (AXI) interfaces, with BRAMs (block RAMs) allocated for weights, biases, and binary p-bit outputs. Weights and biases have fixed point s{6}{3} precision where 's' denotes the sign bit and the first and second curly braces represent the integer and fractional parts.

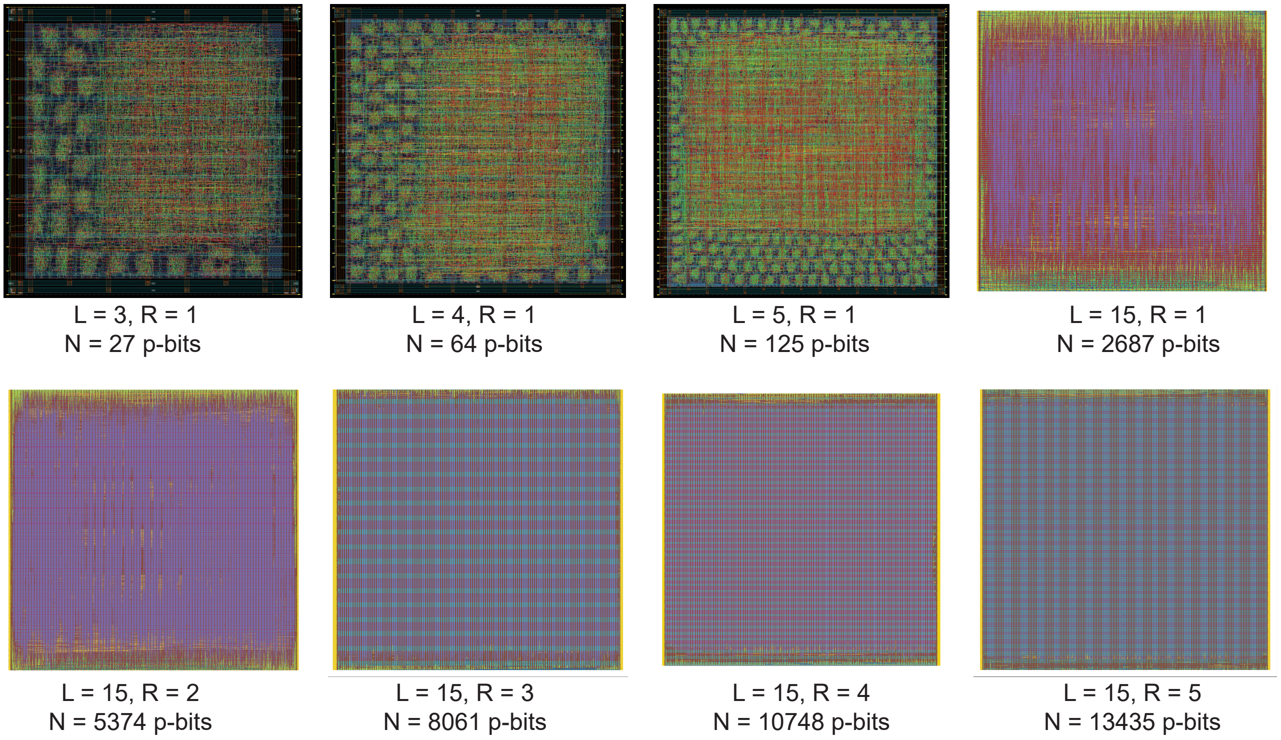

FIG. S10. **Physical design flow results for the DT-SQA algorithm at various scales:** The feasibility of ASIC implementation for the DT-SQA algorithm is evaluated by running the mflowgen physical design flow with the ASAP7 7 nm PDK on custom RTL designs. Results are shown for different combinations of cube size (L) and Trotter replicas (R).

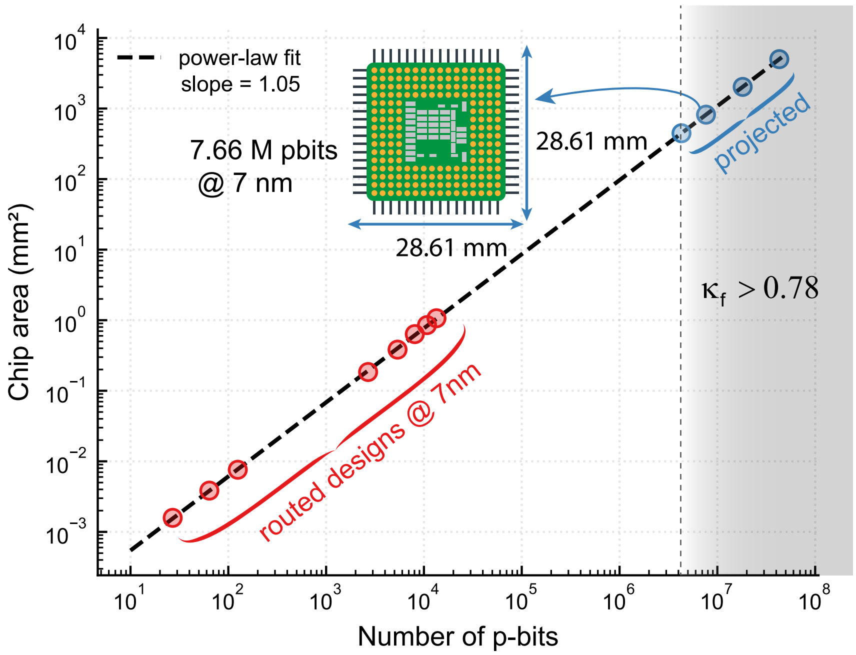

FIG. S11. **Scaling of chip area with the number of p-bits for the DT-SQA algorithm:** The growth in chip area is studied as more p-bits are integrated into the chip, following a full place-and-route design process. The observed trend exhibits near-linear growth, with a slope of 1.05. The largest routed designs, based on the 7 nm ASAP7 PDK, are shown in red, while the projected scaling is represented in blue. Extrapolating to a modern chip size of 28.61×28.61 mm^2, it is estimated that such a chip could accommodate approximately 7.66 million p-bits. This corresponds to 2850 replicas of size $15 \times 15 \times 12$, achieving scaling comparable to a quantum annealer with $\kappa_f > 0.785$.

Algorithm S2: Adaptive parallel tempering with p-computers

Input: Weights, biases, number of swaps, sweeps per swap, colormap, step rate α, initial inverse temperature β_0, energy variance tolerance, number of chains, sweeps per chain

Output: State corresponding to minimum energy, m_{opt}

Function `p-computer` (*weights, biases, colormap, temp.*) **:**
- **for** *each color in the colormap* **do**
  - **for** *each p-bit in the color* **do**
    - Solve Eq. (5) and Eq. (6).

Function `ICMop` (*replica1, replica2*) **:**
- Find the overlap vector between replica 1 and replica 2.
- Randomly pick one cluster where overlap is -1.
- **if** *size of the cluster is greater than half of the total number of spins* **then**
  - Randomly chose one of the two replicas.
  - Flip all the spins of the chosen replica.
- **else**
  - Flip all the spins inside the chosen cluster of replica 1 and replica 2.
- **return** replica 1, replica 2

$t \leftarrow 0$, $\beta_t \leftarrow \beta_0$

Initialize all parallel chains to random states.

while *energy variance is greater than tolerance* **do**
- **for** *each chain in parallel* **do**
  - **for** *each sweep* **do**
    - Sample p-bit states from p-computer.
    - Compute energy of the chain.
  - Compute energy variance for the chain.
  - Save the p-bit states.
- Compute mean energy variance of chains, σ_{E}.
- Set next step inverse temperature: $\beta_{t+1} \leftarrow \beta_t + \dfrac{\alpha}{\sigma_{\text{E}}}$, $t \leftarrow t+1$.

Initialize all replicas to random states

for *each swap attempt* **do**
- **if** *it is an even numbered swap attempt* **then**
  - Choose (even, odd) sequential pairs.
- **else**
  - Choose (odd, even) sequential pairs.
- **for** *each replica in parallel do* **do**
  - **for** *each sweep* **do**
    - Sample p-bit states from p-computer.
  - Compute energy of the replica.
  - Randomly partition ICM replicas into pairs.
  - **for** *each ICM replica pair* **do**
    - Perform ICMop on the replicas in the pair.
- **for** *each sequential pair of replicas* **do**
  - Propose a swap.
  - **if** *accepted* **then**
    - Swap the p-bit states of all corresponding iso-temperature replicas between two replicas.

return p-bit states for the replica with the minimum energy.

the algorithm.

B. Improvement in the performance of APT algorithm with ICM

Here, we justify the inclusion of ICM and compare the performance of APT with and without ICM, as shown in Fig. S14(a). Despite the additional computational overhead introduced by ICM, it offers several advantages: (1) It achieves lower residual

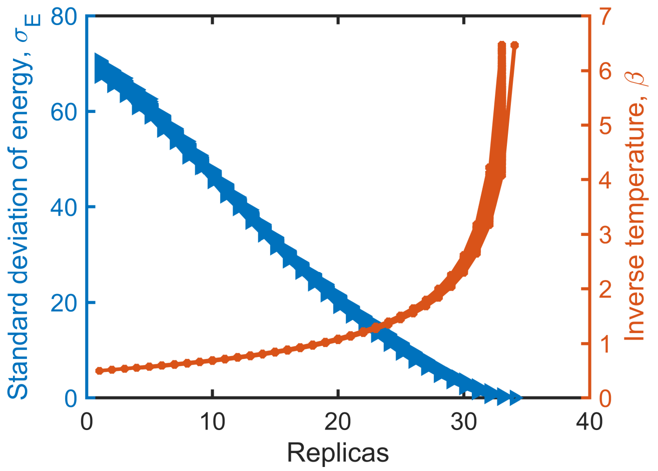

FIG. S12. **Inverse temperature profile of the APT algorithm:** The inverse temperature (β) profiles generated by the preprocessing algorithm for each of the 300 instances are shown. At high temperatures (low β), the profiles across instances are nearly identical, with slight deviations appearing at low temperatures (high β). The figure also shows the standard deviation of sampled energies ($\sigma_{\rm E}$) at each temperature, which decreases monotonically as β increases. The preprocessing begins with $\beta = 0.5$, below which the standard deviation saturates to a constant value. The preprocessing step terminates when the average standard deviation of energy drops below the minimum coupling value, min(J_{ij}).

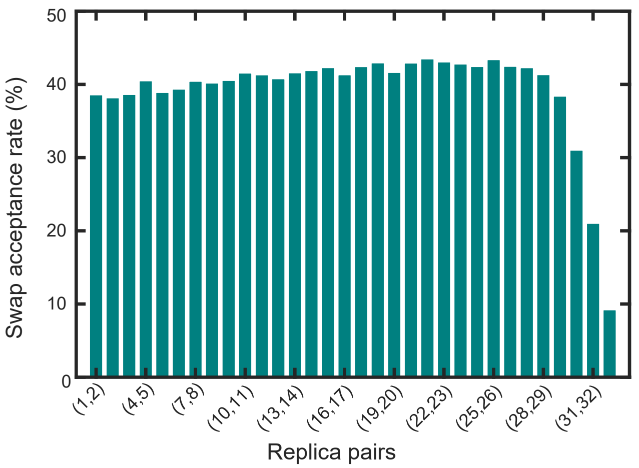

FIG. S13. **Swap acceptance rate of the APT algorithm:** The swap acceptance rate obtained from the APT with ICM algorithm for a randomly chosen instance using the preprocessed temperature profiles is shown. With $\alpha = 1.25$, the average swap acceptance rate across all nearest-neighbor replica pairs is approximately 40%. The results are averaged over 10000 swaps and six independent runs. 4 ICM replicas are used, but their averages are reported as a single replica.

energy compared to APT without ICM for a fixed MCS budget, with the difference becoming more pronounced at larger $t_{\rm a}$ values. (2) It delivers an improved slope compared to APT without ICM. Adding more ICM replicas improves the performance of the algorithm as shown in Fig. S14(b).

A logical question that arises is whether the bending observed in APT with ICM is primarily due to the increased number of replicas or the inclusion of ICM. Supplementary Fig. S15 addresses this by comparing APT with and without ICM. The impact of ICM is evident from the clear separation between the cases: APT using ICM (blue circles and green squares) show lower residual energy compared to those without ICM (purple triangles), even for the same number of replicas. Additionally, performance improvements are observed as the number of replicas increases, further contributing to the bending. This emphasizes the critical role of non-local moves in classical/probabilistic algorithms, which can significantly enhance performance. A natural next step could be to use non-equilibrium Monte Carlo algorithms aimed to improve the APT algorithm [15], but we do not attempt this here.

C. APT with ICM as a function of sweep to swap ratio

Next, we evaluate the performance of APT with ICM as a function of the sweep-to-swap ratio, which defines the number of sweeps performed before each swap attempt. Our findings indicate that the sweep-to-swap ratio significantly impacts the algorithm performance. While a lower sweep-to-swap ratio increases the number of swaps, it consistently results in better residual energy for a fixed MCS budget, as shown in Fig. S16 for three different sweep-to-swap ratios. Replica energies are calculated at each swap attempt and not saved for the entire annealing time. The sweep-to-swap ratio of 1 gives the best residual energy for an MCS budget even though a more typical sweep-to-swap ratio of 10 achieves close performance with a similar bending behavior, therefore this sweep-to-swap choice does not critically change our results. We carefully verified that computing replica energy at each sweep does not affect the conclusion, confirming that the superior performance at a sweep-to-swap ratio of 1 is not simply a result of increased computational effort. The additional computational cost from more frequent swaps can be mitigated using dedicated hardware, as described in the Methods section of the main text.

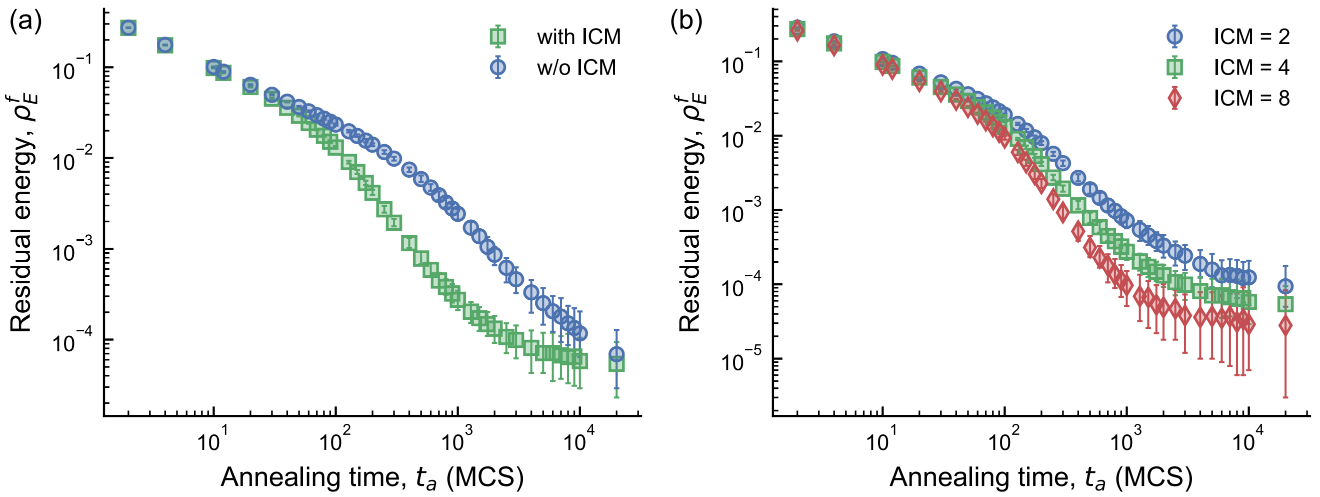

FIG. S14. **Residual energy ($\rho_{\rm E}^{\rm f}$) from APT with and without ICM:** (a) The residual energy as a function of annealing time ($t_{\rm a}$) is shown for the APT algorithm, both with and without isoenergetic cluster moves (ICM) and for cube size $L = 8$ (512 spins). The results demonstrate that incorporating ICM improves performance, yielding lower residual energy compared to APT without ICM, particularly at longer annealing times. In this analysis, one Monte Carlo sweep is performed for each replica before a swap is attempted and 4 ICM replicas are used. (b) The impact of varying the number of ICM replicas is illustrated, with performance improving as the number of ICM replicas increases. Error bars denote 95% bootstrapped confidence interval of mean over 150 instances.

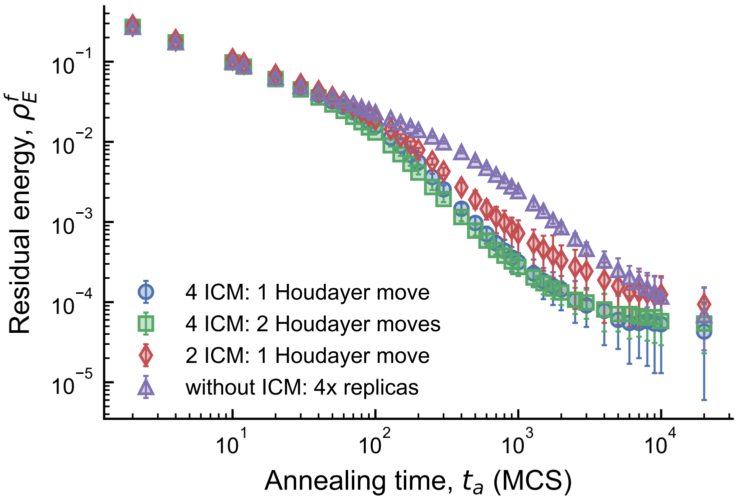

FIG. S15. **Effect of ICM on residual energy ($\rho_{\rm E}^{\rm f}$) in APT with ICM algorithm:** The performance of APT with ICM is compared with varying ICM per MCS to investigate the role of ICM for cube size $L = 8$. A Houdayer move [16] is an isoenergetic cluster update used in spin-glass Monte Carlo simulations. It operates by identifying a cluster of antiparallel spins between two replicas and swapping them to enhance mixing and improve sampling efficiency. With 4 replicas for ICM, two replica pairs are available for ICM–one Houdayer move on each pair. The purple triangles represent APT without any ICM. The blue circles use one ICM on a randomly chosen pair, and the green squares use two ICMs. These three plots use the same number of replicas for a fair comparison. For comparison, we also show APT with 2 ICM replicas (red diamonds) which has only one replica pair for ICM. Error bars denote 95% bootstrapped confidence interval of mean over 150 instances.

D. Performance of APT with ICM on the embedded instances

Fig. S17 compares the performance of APT with ICM on the embedded instances. The performance is very similar to the logical instances showing the transition from a gentler to steeper slope.

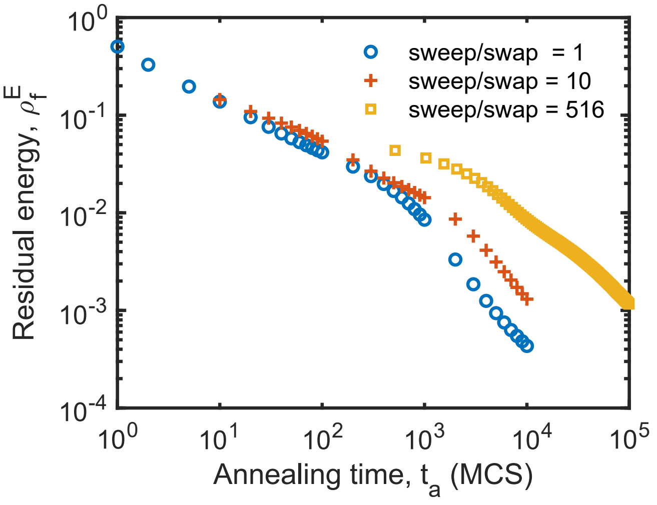

FIG. S16. **Residual energy ($\rho_{\mathrm{E}}^{\mathrm{f}}$) with APT + ICM as a function of sweep-to-swap ratio:** The performance of the APT with ICM algorithm is evaluated for various sweep-to-swap ratios (defined as the number of Monte carlo sweeps performed for each replica before a swap is attempted). The residual energy is plotted as a function of annealing time (t_{a}) for sweep-to-swap ratios of 1, 10, and 516. The results show that a lower sweep-to-swap ratio (sweep/swap = 1) yields the best performance, achieving lower residual energy and a steeper slope, indicating faster convergence towards solutions. In this analysis, 4 ICM replicas are used for ICM.

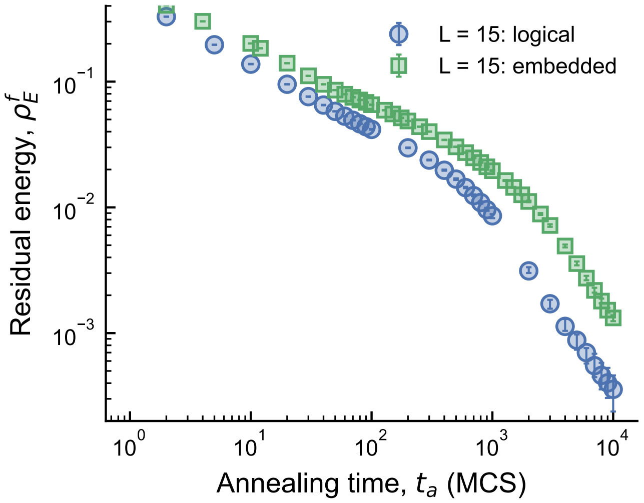

FIG. S17. **Residual energy ($\rho_{\mathrm{E}}^{\mathrm{f}}$) with APT + ICM for embedded instances:** The performance of the APT with ICM algorithm is shown for embedded instances. The residual energy is plotted as a function of annealing time (t_{a}) for sweep-to-swap ratios of 1 and 4 ICM replicas compared with logical instances of the same size ($L = 15$). The embedded instances show similar characteristics to those of logical instances. Error bars denote 95% bootstrapped confidence interval of mean over 300 instances.

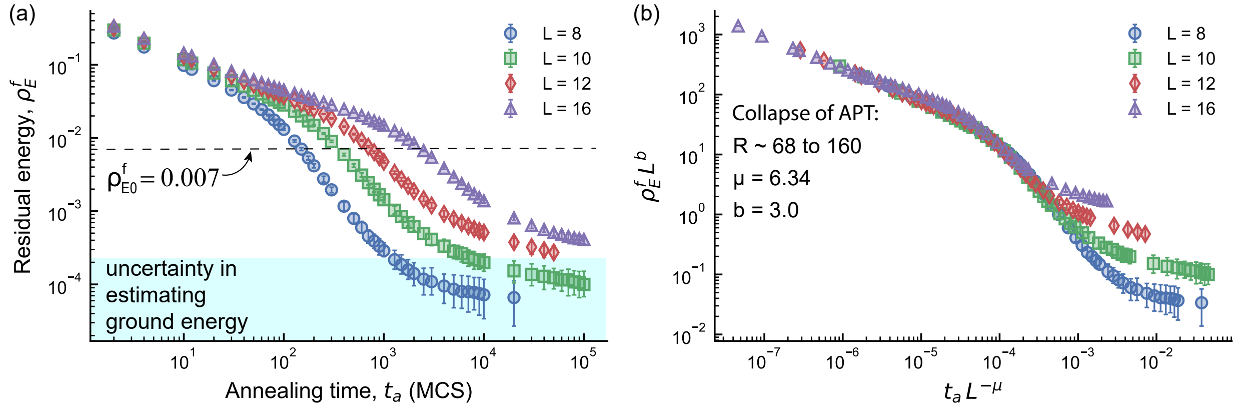

FIG. S18. **Residual energy vs. annealing time t_a plots as a function of system size L of the logical instances for APT with ICM:** (a) Residual energies plotted against the annealing time for a few system sizes L. All data points are averaged over 100 initial conditions per each of the 300 instances provided/generated. (b) The collapse of the residual energy versus annealing time is shown, using the finite size scaling method. Setting $\mu = 6.34$, and $b = 3$ provides a gentle collapse of the data onto a single universal curve although the collapse breaks down at very low residual energies near the ground states of the instances, probably due to the uncertainty in the determination of the ground energy. The $L = 15$ data is excluded from this collapse because it does not correspond to an exact cube. Error bars denote 95% bootstrapped confidence interval of mean over 300 instances.

E. Slope of the residual energy as a function of system size of the logical instances

In Supplementary Fig. S18, we show the final residual energy as a function of annealing time for four different system sizes, for the APT with ICM algorithm. We perform a scaling analysis: the annealing time is rescaled as $t_{\mathrm{a}}L^{-\mu}$, and the residual energy is rescaled as $L^b \rho_{\mathrm{E}}^{\mathrm{f}}$. This rescaling collapses the data onto a single universal curve, indicating that the system behavior follows a universal finite-size scaling law. The $L = 15$ data is excluded from the collapse analysis because these instances do not form a perfect cube ($15 \times 15 \times 12$). At very low residual energies we also observe another transition to a gentler slope, probably due to the uncertainty in the determination of the ground energy (see Methods section of the main text). We define an

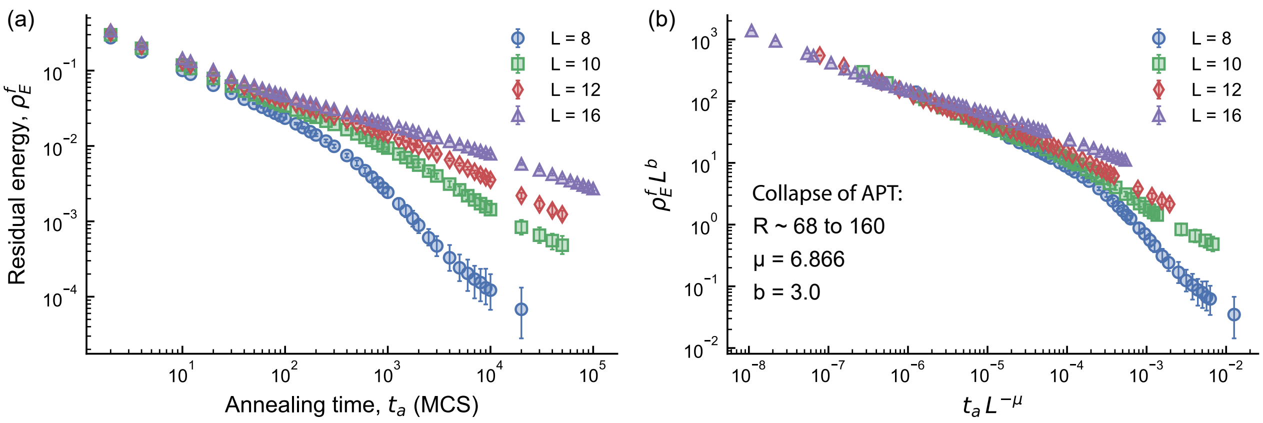

FIG. S19. **Residual energy vs. annealing time t_a plots as a function of system size L of the logical instances for APT without ICM:** (a) Residual energies plotted against the annealing time for a few system sizes L. All data points are averaged over 100 initial conditions per each of the 150 instances provided/generated. (b) The collapse of the residual energy versus annealing time is shown, using the finite size scaling method. Setting $\mu = 6.866$, and $b = 3$ provides a gentle collapse of the early part of the data onto a single universal curve. The later part of the data does not show a second bending like the universal curve for the APT with ICM algorithm. Error bars denote 95% bootstrapped confidence interval of mean over 150 instances.

arbitrarily chosen target residual energy, ρ_{E0}^f, to approximate the optimization performance. For larger system sizes, achieving a given residual energy target requires progressively longer annealing times. The observed universal collapse confirms that the annealing time needed to reach any target residual energy can be predicted approximately for any system size. Supplementary Fig. S19 shows similar plots to those in Supplementary Fig. S18, but for the APT algorithm without ICM. To ensure a fair comparison, the number of replicas is kept consistent with that used in the APT with ICM algorithm. Unlike the case with ICM, the collapse here does not exhibit a second bending.

V. FPGA IMPLEMENTATION OF ADAPTIVE PARALLEL TEMPERING

To evaluate hardware acceleration, we implemented the APT with ICM algorithm on a moderately sized FPGA capable of supporting approximately 5000 p-bits. For system size $L = 15$, the FPGA results (Supplementary Fig. S20) closely match CPU simulations, verifying the correctness of the implementation. The details of the implementation are provided in the Methods section. At this scale, implementation of APT without ICM requires 32 to 34 replicas, while APT with ICM requires 128 to 136 replicas. To overcome FPGA resource constraints, we employ time-division multiplexing (TDM), allowing the same hardware to be reused for multiple replicas. However, this introduces communication overhead, primarily from saving and reloading p-bit states and performing off-chip energy calculations. As a result, FPGA experiments are limited to 10 instances, 10 runs, and a maximum of 1000 MCS. We emphasize that this is not a fundamental limitation. As our hardware feasibility analysis with custom integrated circuits shows, larger FPGAs or ASICs could eliminate the need for TDM. In addition, energy calculations required for the PT swaps can be performed on chip, reducing overhead and improving scalability. Currently, our FPGA setup can accommodate only one replica of size $15 \times 15 \times 12$ (2687 p-bits) or $16 \times 16 \times 16$ (4096 p-bits). The architecture utilizes graph coloring to maximize parallelism, achieving one sweep per replica in 22.22 ns (45 MHz clock; see Methods Section), corresponding to 120 and 185 flips per ns for the respective problem sizes. Performance can be further improved by increasing the number of on-chip p-bits or interconnecting multiple chips. Our implementation achieves a flips per ns metric 50 to 75 times higher than the 2.5 flips per ns reported for optimized simulated annealing on CPUs [11].

REFERENCES

[1] Kerem Y. Camsari, Shuvro Chowdhury, and Supriyo Datta. Scalable Emulation of Sign-Problem–Free Hamiltonians with Room-Temperature p-bits. *Phys. Rev. Applied*, 12:034061, 09 2019.

[2] Giuseppe E. Santoro, Roman Martoňák, Erio Tosatti, and Roberto Car. Theory of Quantum Annealing of an Ising Spin Glass. *Science*, 295(5564):2427–2430, 2002.

[3] Bettina Heim, Troels F. Rønnow, Sergei V. Isakov, and Matthias Troyer. Quantum versus classical annealing of Ising spin glasses. *Science*, 348(6231):215–217, 2015.

[4] H. Rieger and N. Kawashima. Application of a continuous time cluster algorithm to the two-dimensional random quantum Ising ferromagnet. *The European Physical Journal B - Condensed Matter and Complex Systems*, 9(2):233–236, May 1999.

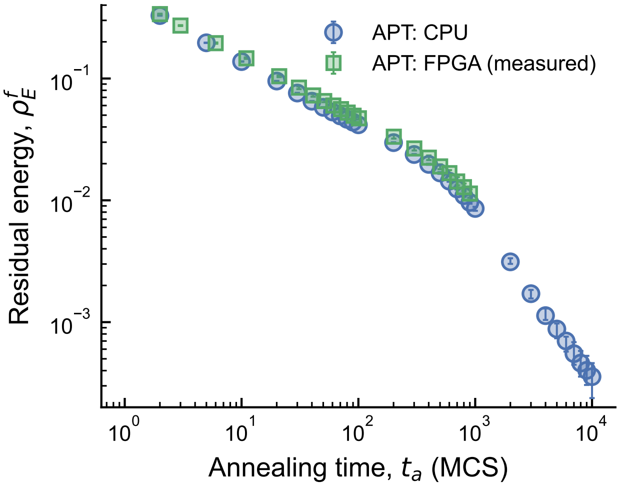

FIG. S20. **Verification of APT with ICM algorithm implemented on FPGA:** Adaptive parallel tempering (APT) with isoenergetic cluster moves (ICM) on CPUs for the problem size $15 \times 15 \times 12$, averaging over 300 instances and using 50 initial conditions per instance. A sweep-to-swap ratio of 1 minimizes residual energy for APT. Also shown FPGA implementation of APT with ICM, running 10 instances with 10 initial conditions each, closely matching the CPU result. Deviations between the FPGA and CPU may be due to the fixed point weight precision used in the FPGA (s{6}{6}, where 's' denotes the sign bit and the first and second curly braces represent the integer and fractional part of the weights, respectively) compared to float64 in CPU and possibly due to the pseudorandom number generator differences (LFSR in the FPGA and Mersenne Twister in the CPU), though this difference typically does not play a significant role. Note that this precision is higher than what we used for DT-SQA in our feasibility analysis, due to the sensitivity of the APT algorithm to the weight precision. Error bars denote 95% bootstrapped confidence interval of mean over instances.

[5] Navid Anjum Aadit, Andrea Grimaldi, Mario Carpentieri, Luke Theogarajan, John M Martinis, Giovanni Finocchio, and Kerem Y Camsari. Massively parallel probabilistic computing with sparse Ising machines. *Nature Electronics*, 5(7):460–468, 2022.

[6] Srijan Nikhar, Sidharth Kannan, Navid Anjum Aadit, Shuvro Chowdhury, and Kerem Y Camsari. All-to-all reconfigurability with sparse and higher-order Ising machines. *Nature Communications*, 15(1):8977, 2024.

[7] Shuvro Chowdhury, Andrea Grimaldi, Navid Anjum Aadit, Shaila Niazi, Masoud Mohseni, Shun Kanai, Hideo Ohno, Shunsuke Fukami, Luke Theogarajan, Giovanni Finocchio, Supriyo Datta, and Kerem Y. Camsari. A Full-Stack View of Probabilistic Computing With p-Bits: Devices, Architectures, and Algorithms. *IEEE Journal on Exploratory Solid-State Computational Devices and Circuits*, 9(1): 1–11, 2023.

[8] M. Mahmudul Hasan Sajeeb, Navid Anjum Aadit, Shuvro Chowdhury, Tong Wu, Cesely Smith, Dhruv Chinmay, Atharva Raut, Kerem Y. Camsari, Corentin Delacour, and Tathagata Srimani. Scalable connectivity for Ising machines: Dense to sparse. *Phys. Rev. Appl.*, 24: 014005, Jul 2025.

[9] William A Borders, Ahmed Z Pervaiz, Shunsuke Fukami, Kerem Y Camsari, Hideo Ohno, and Supriyo Datta. Integer factorization using stochastic magnetic tunnel junctions. *Nature*, 573(7774):390–393, 2019.

[10] Troels F. Rønnow, Zhihui Wang, Joshua Job, Sergio Boixo, Sergei V. Isakov, David Wecker, John M. Martinis, Daniel A. Lidar, and Matthias Troyer. Defining and detecting quantum speedup. *Science*, 345(6195):420–424, July 2014.

[11] Andrew D. King, Jack Raymond, Trevor Lanting, Richard Harris, Alex Zucca, Fabio Altomare, Andrew J. Berkley, Kelly Boothby, Sara Ejtemaee, Colin Enderud, Emile Hoskinson, Shuiyuan Huang, Eric Ladizinsky, Allison J. R. MacDonald, Gaelen Marsden, Reza Molavi, Travis Oh, Gabriel Poulin-Lamarre, Mauricio Reis, Chris Rich, Yuki Sato, Nicholas Tsai, Mark Volkmann, Jed D. Whittaker, Jason Yao, Anders W. Sandvik, and Mohammad H. Amin. Quantum critical dynamics in a 5,000-qubit programmable spin glass. *Nature*, 617(7959): 61–66, April 2023. ISSN 1476-4687.

[12] Andrew D. King, Sei Suzuki, Jack Raymond, Alex Zucca, Trevor Lanting, Fabio Altomare, Andrew J. Berkley, Sara Ejtemaee, Emile Hoskinson, Shuiyuan Huang, Eric Ladizinsky, Allison J. R. MacDonald, Gaelen Marsden, Travis Oh, Gabriel Poulin-Lamarre, Mauricio Reis, Chris Rich, Yuki Sato, Jed D. Whittaker, Jason Yao, Richard Harris, Daniel A. Lidar, Hidetoshi Nishimori, and Mohammad H. Amin. Coherent quantum annealing in a programmable 2,000 qubit Ising chain. *Nature Physics*, 18(11):1324–1328, November 2022. ISSN 1745-2481. Number: 11 Publisher: Nature Publishing Group.

[13] Alex Carsello, James Thomas, Ankita Nayak, Po-Han Chen, Mark Horowitz, Priyanka Raina, and Christopher Torng. mflowgen: a modular flow generator and ecosystem for community-driven physical design: invited. In *Proceedings of the 59th ACM/IEEE Design Automation Conference*, DAC '22, page 1339–1342, New York, NY, USA, 2022. Association for Computing Machinery. ISBN 9781450391429.

[14] Lawrence T. Clark, Vinay Vashishtha, Lucian Shifren, Aditya Gujja, Saurabh Sinha, Brian Cline, Chandarasekaran Ramamurthy, and Greg Yeric. ASAP7: A 7-nm finFET predictive process design kit. *Microelectronics Journal*, 53:105–115, 2016. ISSN 1879-2391.

[15] Masoud Mohseni, Daniel Eppens, Johan Strumpfer, Raffaele Marino, Vasil Denchev, Alan K. Ho, Sergei V. Isakov, Sergio Boixo, Federico Ricci-Tersenghi, and Hartmut Neven. Nonequilibrium Monte Carlo for Unfreezing Variables in Hard Combinatorial Optimization. *arXiv*, nov 2021.

[16] J. Houdayer. A cluster Monte Carlo algorithm for 2-dimensional spin glasses. *The European Physical Journal B*, 22(4):479–484, August 2001. ISSN 1434-6028.
